# Quantitative detection of α-Synuclein and Tau oligomers and other aggregates by digital single particle counting

**DOI:** 10.1038/s41531-022-00330-x

**Published:** 2022-06-02

**Authors:** Lara Blömeke, Marlene Pils, Victoria Kraemer-Schulien, Alexandra Dybala, Anja Schaffrath, Andreas Kulawik, Fabian Rehn, Anneliese Cousin, Volker Nischwitz, Johannes Willbold, Rebecca Zack, Thomas F. Tropea, Tuyen Bujnicki, Gültekin Tamgüney, Daniel Weintraub, David Irwin, Murray Grossman, David A. Wolk, John Q. Trojanowski, Oliver Bannach, Alice Chen-Plotkin, Dieter Willbold

**Affiliations:** 1 Institute of Biological Information Processing (Structural Biochemistry: IBI-7), Forschungszentrum Jülich, 52428 Jülich, Germany; 2 attyloid GmbH, 40225 Düsseldorf, Germany; 3 Institut für Physikalische Biologie, Heinrich-Heine-Universität Düsseldorf, 40225 Düsseldorf, Germany; 4 Central Institute for Engineering, Electronics and Analytics, Analytics (ZEA-3), Forschungszentrum Jülich, 52428 Jülich, Germany; 5 Department of Neurology, Perelman School of Medicine, University of Pennsylvania, Philadelphia, PA USA; 6 Center for Neurodegenerative Disease Research, Perelman School of Medicine, University of Pennsylvania, Philadelphia, PA USA; 7 Department of Psychiatry, Perelman School of Medicine, University of Pennsylvania, Philadelphia, PA USA; 8 Parkinson’s Disease and Mental Illness Research, Education, and Clinical Centers, Philadelphia Veterans Affairs Medical Center, Philadelphia, PA USA; 9 Department of Pathology and Laboratory Medicine, Perelman School of Medicine, University of Pennsylvania, Philadelphia, PA USA

**Keywords:** Diagnostic markers, Parkinson's disease, Diagnostic markers

## Abstract

The pathological hallmark of neurodegenerative diseases is the formation of toxic oligomers by proteins such as alpha-synuclein (aSyn) or microtubule-associated protein tau (Tau). Consequently, such oligomers are promising biomarker candidates for diagnostics as well as drug development. However, measuring oligomers and other aggregates in human biofluids is still challenging as extreme sensitivity and specificity are required. We previously developed surface-based fluorescence intensity distribution analysis (sFIDA) featuring single-particle sensitivity and absolute specificity for aggregates. In this work, we measured aSyn and Tau aggregate concentrations of 237 cerebrospinal fluid (CSF) samples from five cohorts: Parkinson’s disease (PD), dementia with Lewy bodies (DLB), Alzheimer’s disease (AD), progressive supranuclear palsy (PSP), and a neurologically-normal control group. aSyn aggregate concentration discriminates PD and DLB patients from normal controls (sensitivity 73%, specificity 65%, area under the receiver operating curve (AUC) 0.68). Tau aggregates were significantly elevated in PSP patients compared to all other groups (sensitivity 87%, specificity 70%, AUC 0.76). Further, we found a tight correlation between aSyn and Tau aggregate titers among all patient cohorts (Pearson coefficient of correlation *r* = 0.81). Our results demonstrate that aSyn and Tau aggregate concentrations measured by sFIDA differentiate neurodegenerative disease diagnostic groups. Moreover, sFIDA-based Tau aggregate measurements might be particularly useful in distinguishing PSP from other parkinsonisms. Finally, our findings suggest that sFIDA can improve pre-clinical and clinical studies by identifying those individuals that will most likely respond to compounds designed to eliminate specific oligomers or to prevent their formation.

## Introduction

**Fig. 1:**
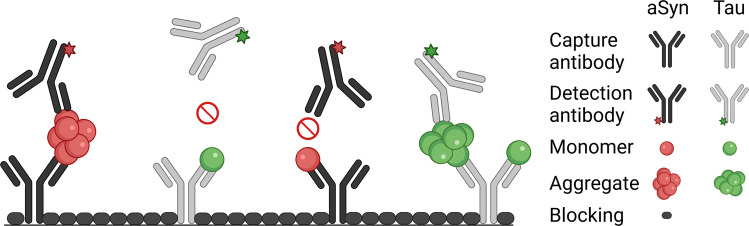
Scheme of the sFIDA assay. Antibodies directed against linear epitopes of aSyn (211) or Tau (Tau5) are immobilized on the glass surface of a microtiter plate. Monomers and aggregates of the sample can bind to the capture antibodies, but only aggregates are detected with fluorescently labeled probes (211 CF633 and Tau5 CF488A) because capture and detection antibodies bind the same epitope. For monomeric protein, this epitope is masked by the capture antibody and can therefore not be bound by a probe antibody. Finally, the assay surface is imaged by dual-color fluorescence microscopy and single particles on the well surface are counted by image-data analysis. *Created with BioRender.com*.

Tauopathies and synucleinopathies are characterized by abnormal aggregation of microtubule-associated protein tau (Tau) and alpha-synuclein (aSyn), respectively. From the clinical perspective there is some overlap in the phenotypic presentation of the resulting diseases, with parkinsonism characterizing multiple diseases, including Parkinson’s disease (PD), dementia with Lewy bodies (DLB), and progressive supranuclear palsy (PSP)^1,2^. While protein aggregation is the pathological key event in these disorders, ultimately resulting in the formation of aSyn and Tau deposits, the neurotoxic effect is thought to be exerted by small oligomeric intermediates within the aggregation pathway^3–5^. Consequently, a number of drug candidates have been designed to interfere with the aggregation pathway aiming to eliminate existing oligomers or to prevent their formation^6^. Since aggregate formation reflects pathophysiological changes inside the brain, oligomers have also been proposed as promising biomarker candidates^7–9^. However, quantitative measurement of oligomers is technically challenging and mainly hampered by three technical issues. First, the minute amount of oligomers in human biofluids such as cerebrospinal fluid (CSF) requires extreme sensitivity. Secondly, the presence of a vast excess of monomers demands high selectivity for oligomers over monomeric species. Quantitation of oligomeric aSyn by ELISA-like techniques, which employ overlapping epitopes or antibody probes directed against structural motifs, render these assays insensitive towards monomers^10^. Our previously developed sFIDA technology (surface-based fluorescence intensity distribution analysis) employs a similar biochemical setup using the same capture and detection antibody (Fig. 1) but features single-particle sensitivity through a microscopy-based readout^11^. Thirdly, the structural diversity of aggregates renders their detection technically challenging^12^. sFIDA uses linear epitopes and therefore detects and counts all subtypes of aggregated protein irrespectively of higher-ordered structures, while assays using structural epitopes only determine a subfraction of oligomers, fibrils, or other aggregates from a heterogeneous pool of structures. Because the assay itself is not yet discriminating between small oligomers and larger, but still soluble assemblies, like protofibrils, seeding competent fibrillar oligomers or fibrils, we refer to the analytes measured by sFIDA as aggregates, irrespectively, whether they are on or off pathway to fibrils^13^.

While our prior work establishes the technical concept of sFIDA^11,14,15^, its utility in clinical samples from neurodegenerative disease patients is yet to be established. In the present work, we apply sFIDA to quantitate aSyn and Tau aggregates in CSF from 237 individuals, demonstrating its applicability in clinical settings and drug development.

## Results

In this work, we have developed an sFIDA assay for simultaneous quantification of aSyn and Tau aggregates. Development and validation of immunoassays require determination of crucial parameters including limit of detection (LOD), coefficient of variation, inter-assay and inter-laboratory correlation and cross reactivity, which are described in the first part of the results chapter.

### sFIDA displays low intra-assay variance for measurements of SiNaPs and samples

**Fig. 2:**
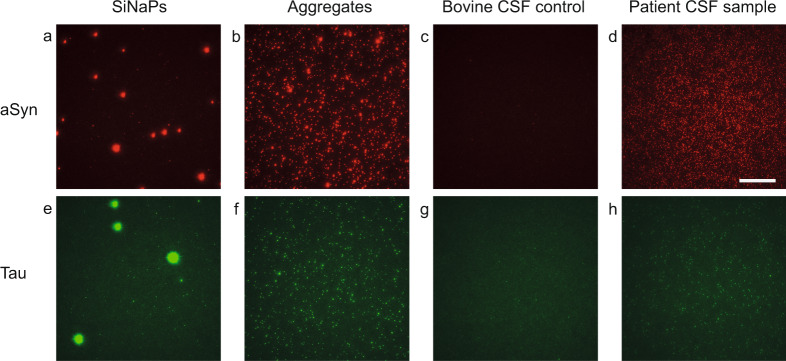
TIRFM images of aSyn and Tau SiNaPs, synthetic aggregates, and samples. Shown are characteristic TIRFM images for the red fluorescence channel (211 CF633) of **a** 629 fM aSyn SiNaPs in buffer, **b** 8 nM aSyn aggregates in buffer (the concentration is based on the monomer concentration), **c** a bovine CSF control, **d** a CSF sample of a PD patient as well as for the green fluorescence channel (Tau5 CF488) of **e** 645 fM Tau SiNaPs in buffer, **f** 200 nM Tau aggregates in buffer (the concentration is based on the monomer concentration), **g** a bovine CSF control, **h** a CSF sample of a PSP patient. The scale bar is 25 µm. For illustration of the 14-bit images, the contrast was adjusted to a maximum grayscale value of 5000.

The sFIDA technology was used to determine the concentrations of aggregated aSyn and aggregated Tau in a total of 237 CSF samples. Due to the high number of assay points, the measurements were performed on a total of eight 384 well microtiter plates. For calibration of the samples and determination of the LOD, we used our previously developed silica nanoparticle (SiNaP) standard^14^ (TEM image and size distribution in Supplementary Fig. 1). Exemplary images of SiNaPs, aggregates, bovine CSF and patient samples are shown in Fig. 2. The intra-assay variance among all experiments was calculated from the pixel counts of the four replicates. The intra-assay variance for the calibration standard was 15.8% for aSyn SiNaPs and 19.1% for Tau SiNaPs for the concentrations included in the calibration range. The intra-assay variance of the samples was 16.8% for aSyn aggregates and 13.0% for Tau aggregates, respectively (individual results for each experiment in Supplementary Table 1).

### Independent measurements of aSyn and Tau aggregates in CSF samples yield comparable results

**Fig. 3:**
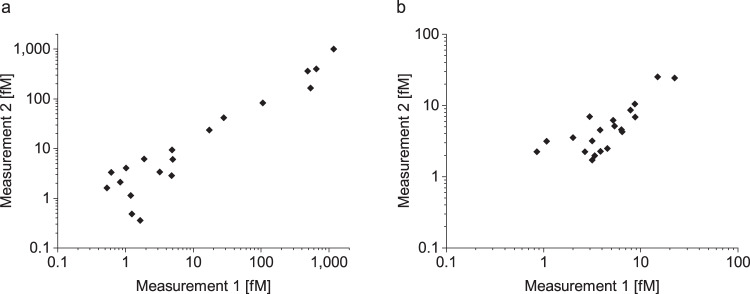
Repeated measurements of aSyn and Tau aggregates in CSF yield highly reproducible results. We tested the inter-assay variance of the sFIDA assay for **a** aSyn and **b** Tau aggregates. Two independent measurements of 20 CSF samples by the same technician in the same laboratory on different days were highly reproducible with a Pearson coefficient of correlation of *r* = 0.96 for aSyn aggregates and *r* = 0.92 for Tau aggregates.

The inter-assay variance was studied in 20 samples on two different runs that were executed at a four-month interval. We analyzed each sample in four replicates and determined an intra-assay variation for the 20 CSF samples of 22.6% for aSyn aggregates and 20.4% for Tau aggregates. A linear correlation between the two measurements was observed for the detection of aSyn aggregates with a Pearson coefficient of correlation of *r* = 0.964. Although the concentration of Tau aggregates was less than that of aSyn aggregates and very close to the LOD, the two measurements showed a significant correlation with a Pearson coefficient of correlation of *r* = 0.920 (Fig. 3).

**Fig. 4:**
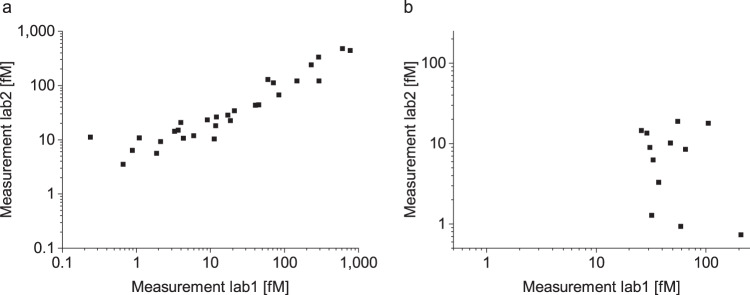
Measurements of aSyn but not Tau aggregates in CSF correlated well when measured in a different laboratory. The CSF samples were measured by sFIDA by two different technicians in two different laboratories. The first sFIDA experiment was prepared and run by a technician at the Forschungszentrum Jülich, while the second sFIDA was performed by another technician at the Heinrich-Heine-Universität Düsseldorf. **a** Concentrations of aSyn aggregates correlated well between both experiments with a Pearson coefficient of correlation of *r* = 0.95, **b** while for Tau aggregates no correlation was observed (*r* = 0.03).

The inter-laboratory variance was studied in a laboratory at the Forschungszentrum Jülich and another laboratory at the Heinrich-Heine-Universität in Düsseldorf. The calibrated results for the detection of aSyn aggregates showed a high correlation with a Pearson coefficient of correlation of *r* = 0.950. In this study, no correlation for Tau aggregates was observed (Pearson coefficient of correlation of *r* = 0.033, Fig. 4).

### sFIDA features femtomolar sensitivity for the detection of aSyn and Tau SiNaPs

We determined the sensitivity of the assay based on aSyn and Tau SiNaPs. After application of t-test, Mann-Whitney U test, LOD and standardization of the calibration range for all experiments, the upper limit of the calibration curve was 1.99 pM for aSyn and 2.04 pM for Tau SiNaPs, and the lower limit was set to 63 fM and 204 fM for aSyn and Tau SiNaPs, respectively. In all experiments a linear correlation between pixel count and concentration with a mean coefficient of determination of 0.98 for aSyn and 0.96 for Tau SiNaPs was observed. The calibration resulted in a mean LOD of 6.72 fM for the detection of aSyn SiNaPs and a mean LOD of 33.7 fM for the detection of Tau SiNaPs (individual LOD values for each experiment are shown in Supplementary Table 2). For aSyn aggregates, 66% of samples were above the LOD of the individual experiment and for Tau aggregates 44% (Supplementary Table 3).

### sFIDA shows negligible cross reactivity for measurements of aSyn, amyloid beta, and Tau

**Fig. 5:**
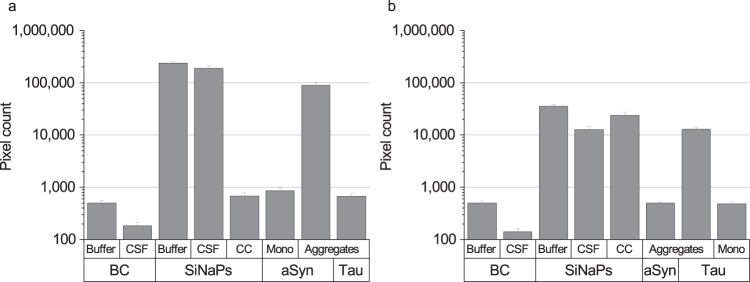
Pixel counts of assay controls for the detection of aSyn and Tau aggregates. **a** aSyn SiNaPs showed a recovery of 79% in CSF, whereas **b** Tau SiNaPs showed a recovery of 36% in CSF. Synthetic aSyn and Tau aggregates served as positive controls. The pixel counts of the aSyn aggregates when detected with the Tau5 antibody as well as the pixel counts of the Tau aggregates when detected with the 211 antibody were as low as the blank control (BC). The pixel count of 8 nM monomeric (Mono) aSyn as well as 200 nM monomeric Tau was reduced by 99.5% compared to the same concentration of monomer units in aggregated aSyn or Tau. When the capture antibody was omitted (capture control, CC), no signal was detected for aSyn SiNaPs, whereas the signal for Tau SiNaPs was still at 67%. Standard deviation was calculated from the four replicates.

To determine the selectivity of the sFIDA assay, amyloid beta SiNaPs with a concentration of 6 pM were used as a control. The amyloid beta SiNaPs were coated with amino acid residues 1–15 of the amyloid beta protein. We observed a very low cross reactivity with 0.1% of the signal for amyloid beta SiNaPs when compared to the signal obtained with aSyn SiNaPs and 0.2% of the signal obtained with Tau SiNaPs when used at a comparable concentration (data not shown). Detection of synthetic Tau aggregates with the 211 antibody and detection of synthetic aSyn aggregates with the Tau5 antibody resulted in a pixel count as negligible as for the buffer control (Fig. 5). The pixel count of the buffer control (BC) showed an increased background signal compared to the CSF control for aSyn (capture and detection antibody: 211) and Tau (capture and detection antibody: Tau5) (Fig. 5). Therefore, the CSF control was used as negative control for the calibration of the samples. The buffer control showed no autofluorescence signal (data not shown). The recovery of aSyn SiNaPs spiked in bovine CSF was 79%. For Tau SiNaPs the recovery in CSF was 36%. Another control was to run the assay without a capture antibody, which is an indication for unspecific binding of silica nanoparticles or proteins to the surface, and is described as the signal compared to the same concentration of silica nanoparticles/protein on an antibody surface. For aSyn, the signal originating from 6 pM silica nanoparticles without the use of a capture antibody was below 0.1% of that when a capture antibody was used. For Tau, the signal was still at 67% without a capture antibody and, presumably, originating from large particles which non-specifically stick to the glass surface. Furthermore, we investigated the effect of monomeric aSyn and Tau on the sFIDA readout.

We found that the pixel counts of both aSyn and Tau monomers, respectively, were decreased by about 99.5% compared to the signal of aSyn or Tau aggregates indicating that endogenous monomers in the CSF samples have a neglectable effect on the sFIDA readout (Fig. 5).

**Fig. 6:**
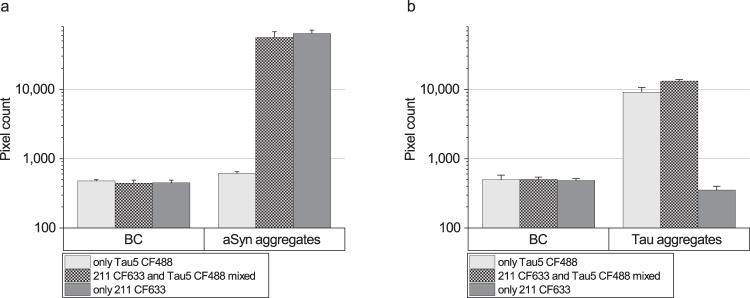
Analysis of probe interference. To analyze the effect of detection probe interference on the signal, we performed an additional experiment in which we added either only one detection antibody or both detection antibodies. Using only the relevant antibody probe did not show an increased pixel count for the detection of aggregated aSyn (**a**, 211 CF633) or Tau (**b**, Tau5 CF488), respectively. Standard deviation was calculated from the four replicates.

To address the question whether addition of the mixture of two antibodies might impair assay sensitivity, we added just the relevant detection antibody or a mixture of both detection antibodies, and compared the correspondent sFIDA readouts. As shown in Fig. 6, applying just a single detection antibody did not increase the readout. Moreover, absence of confounding autofluorescence signals was demonstrated, because aggregated aSyn and Tau did not show any non-specific signal when the relevant antibody probe was not applied.

### aSyn and Tau aggregates are removed by immunodepletion

**Fig. 7:**
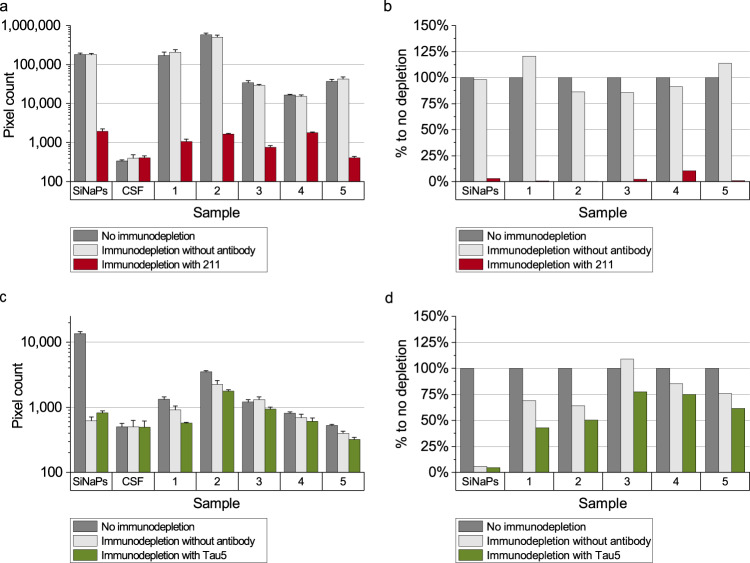
Immunodepletion of aSyn and Tau in CSF samples. The SiNaP standard and five CSF samples were subjected to immunodepletion with magnetic beads with and without antibody. **a**, **b** Immunodepletion of samples with 211 antibody decreased the pixel counts for aSyn on average by 97%, while incubation with magnetic beads without antibody did not affect the signal. **c**, **d** For aggregated Tau, incubation with magnetic beads without antibody decreased the pixel count by 94% for Tau SiNaPs and on average by 19.5% for samples. Using Tau5 antibody, the decrease of the pixel count for Tau SiNaPs was 95% and for samples 38.6%, respectively. The *% to no depletion* value (Fig. 7 **b**, **d**) was calculated by the ratio the pixel counts of depleted to non-depleted samples. Standard deviation was calculated from the four replicates.

To show that the signal measured by sFIDA is specifically attributed to aSyn and Tau aggregates and not to matrix interference, we performed immunodepletion in five CSF samples and the silica nanoparticle standard. To remove the analytes, samples were incubated in presence of magnetic beads linked to 211 antibody, Tau5 antibody or no antibody. After magnetic separation, the supernatants were subjected to sFIDA analysis. For aSyn, 211-depleted samples showed a mean decrease of the readout by 97.0% (Fig. 7b), while in the controls without 211 antibody, the readout was not reduced (−0.6%). Although the readout for Tau aggregates in the samples was comparatively low and close to that of the bovine CSF control, depletion with Tau5 decreased the readout by 38.6% (Fig. 7c, d). Incubation of the samples with magnetic beads alone led to an average decrease of the pixel count by only 19.5% (Fig. 7d). Still, the less efficient reduction in the samples compared to the standards can be attributed to a lower signal-to-noise ratio. The Tau-coated silica nanoparticle standard was depleted even without Tau5 antibody, suggesting non-specific adherence to the surface as observed in the capture control experiment (Fig. 5b).

### Analysis of potential heterophilic anti-mouse antibodies (HAMAs) interference

**Fig. 8:**
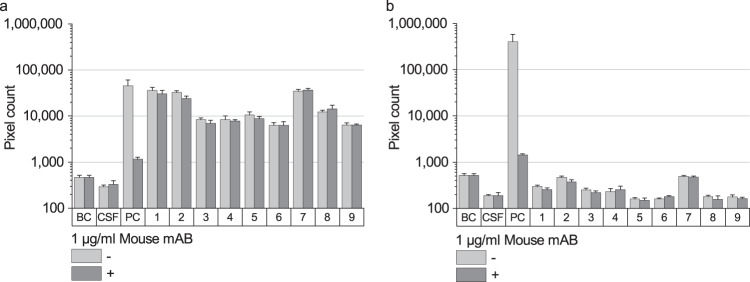
Influence of heterophilic anti-mouse antibodies. To test, if the signal obtained by sFIDA was possibly caused by heterophilic anti-mouse antibodies, nine CSF samples were incubated with or without 1 µg/ml MOPC-21. As a HAMA model and positive control (PC), we used a goat anti-mouse antibody. The interference of this anti-mouse antibody was reduced by about 98.4% for aSyn (211 CF633, **a**) and 99.7% for Tau (Tau5 CF488, **b**) when adding MOPC-21 while for the CSF samples the signal remains unaffected. Standard deviation was calculated from the four replicates.

Although one may expect absence of high antibody titers in CSF, we investigated whether HAMAs could possibly compromise the sFIDA assay result, and whether this is potentially relevant for the interpretation of the study results. HAMAs are a well-described interfering factor in immunoassays especially for blood-based assays^16,17^. HAMAs can crosslink capture and detection mouse antibodies leading to false positive signals. Recent research indicates, that HAMAs can also interfere with measurements of CSF samples^18,19^. To analyze HAMA interference in our assay, we used an anti-mouse antibody as a HAMA model. As expected, sFIDA analysis of a blank control spiked with the anti-mouse antibody shows an increased signal in both detection channels (Fig. 8, PC). Addition of the same concentration of a competitor mouse antibody (MOPC-21) reduces the signal by 98.4% for 211 CF633 and by 99.7% for Tau5 CF488. Additionally, we tested nine CSF samples, which yielded high sFIDA readouts, for possible presence of HAMAs. Incubation of the samples with MOPC did not influence sFIDA readouts (p-value of two-sided Mann-Whitney-U for 211 CF633: 0.470, Tau5 CF488: 0.800) suggesting that the observed signal indeed originates from aggregate-bound probes and is not due to HAMA interference.

### Contamination with blood did not affect the quantification of aSyn and Tau aggregates in CSF

As blood shows an increased concentration of total aSyn, the contamination of CSF even with low amounts of blood can interfere with the detection of aSyn monomers in CSF^20,21^. To investigate, whether the results in sFIDA were affected by blood contamination as well, we classified the samples into five groups according to their contamination level. Most of the samples (57%) showed no contamination with blood (negative test result), 11% were classified to contamination level 1 (~10 Ery/µL), 7% to level 2 (~25 Ery/µL), and 8% to level 3 (~50 Ery/µl). A contamination of level 4 (> 250 Ery/µL) was observed in 18% of CSF. We further investigated, whether there is a correlation between high readouts in sFIDA and the blood contamination level. We could not observe a significant increase of aSyn (*p* = 0.776) or Tau (*p* = 0.628) aggregate concentrations in CSF contaminated with blood using Kruskal-Wallis ANOVA (scatterplot in Supplementary Fig. 2). Therefore, no samples were excluded from analysis.

### Descriptive analysis of the patient and control cohorts

The samples comprised five diagnostic groups (Table 1). Applying the Kruskal-Wallis test, no significant differences between groups was found for age and gender. For education, PD patients received longer education than AD patients, and normal controls received longer education than the AD and PSP cohorts. Individual information and results of each patient are listed in Supplementary Table 4.

**Table 1:** Demographic information on patients and controls that donated CSF samples.

	PD	AD	DLB	PSP	*N*
Number	115	28	19	30	45
Female	41 (36%)	10 (36%)	5 (26%)	15 (50%)	20 (44%)
Age [years]	65.7 (±7.6)	68.2 (±6.3)	69.7 (±7.2)	67.5 (±6.2)	69.0 (±8.9)
Education [years]	16.3 (±2.3)	14.9 (±3.2)	15.5 (±2.8)	15.2 (±2.7)	16.6 (±3.6)
Deceased	17%	32%	32%	37%	13%

*PD* Parkinson’s disease, *AD* Alzheimer’s disease, *DLB* Dementia with Lewy bodies, *PSP* Progressive supranuclear palsy, *N* Normal control.

### aSyn and Tau aggregate levels distinguish patients with different neurodegenerative diseases

**Fig. 9:**
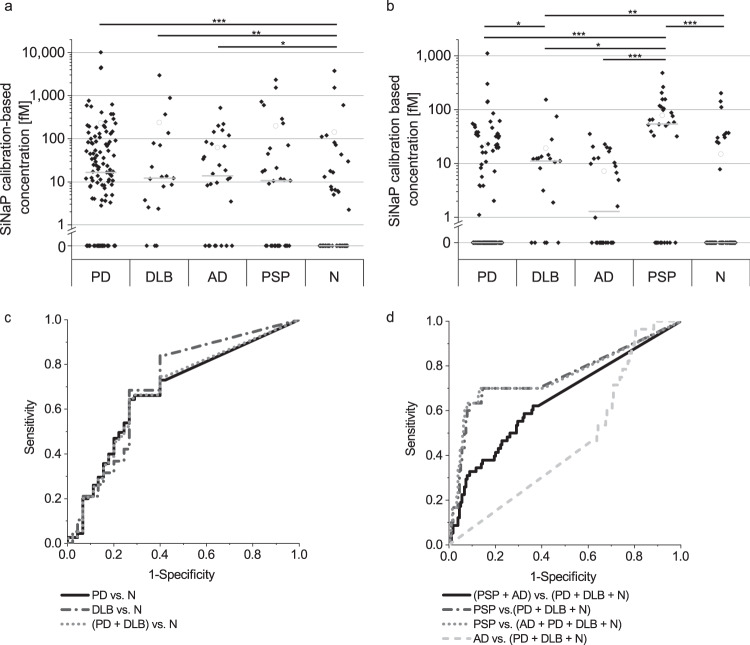
Calibrated sFIDA results (a, b) and receiver operating characteristic (ROC) analysis (c, d) for the detection of aSyn and Tau aggregates in CSF samples. **a** For aSyn aggregates, PD, DLB, and AD samples were significantly elevated compared to normal controls (N). **c** In ROC analysis, discrimination of PD patients versus normal controls (N) showed a specificity of 73% and a sensitivity of 64% with an AUC of 0.68, while discrimination of DLB patients versus normal controls showed a specificity of 60% and a sensitivity of 84% with an AUC of 0.71. In combination, synucleinopathies (PD and DLB) can be differentiated from normal controls with a specificity of 73% and a sensitivity of 65% with an AUC of 0.68. **b**, **d** For Tau aggregates, the tauopathy PSP but not AD can be discriminated from non-tauopathies (for PSP vs. non-tauopathies: 86% specificity and 70% sensitivity with an AUC of 0.75; for other specificity and sensitivity values s. Table 3). DLB samples showed significantly increased Tau aggregate concentrations compared to normal controls and PD patients (*p*-values of Mann-Whitney U test for aSyn and Tau aggregates are shown in Table 2). Values below the LOD were set to 0. “-“ indicates the median and “○” the mean. Significant differences between cohorts were calculated with Mann-Whitney U test and signed with * (*p = 0.01–0.05; **p = 0.001–0.01; ***p < 0.001). Please, note the logarithmic concentration scales.

First, we tested the calibrated results of all groups for normal distribution. As the data showed non-normal distributions (*p*-value < 0.05, Supplementary Table 5), statistical analysis was performed using non-parametric tests like the Kruskal-Wallis or Mann-Whitney U test. The results of the Kruskal-Wallis test showed significant differences between the diagnostic groups for aggregated aSyn (*p* = 6.92*10^−3^) as well as for aggregated Tau (*p* = 2.17*10^−6^). The results of pairwise comparisons are shown in Table 2. Concentrations of aSyn aggregates in CSF samples of PD patients were significantly increased compared to the control group. Moreover, patients with DLB showed elevated levels of aSyn aggregates in their CSF. Interestingly, CSF samples of AD patients also showed significantly increased levels of aSyn aggregates compared to normal controls. In the scatterplot (Fig. 9a) as well as in the receiver operating characteristic (ROC) curve (Fig. 9c) we observed a great overlap between synucleinopathies like PD and DLB and the control group (sensitivity and specificity values and AUC in Table 3).

**Table 2:** *P*-values of two-sided Mann-Whitney U test for pairwise comparisons of measured aSyn and of Tau aggregate concentrations.

		PD	DLB	PSP	AD	*N*
aSyn	PD	1				
	DLB	0.992	1			
	PSP	0.292	0.326	1		
	AD	0.811	0.887	0.561	1	
	N	3.6*10^−4^	0.007	0.109	0.010	1
Tau	PD	1				
	DLB	0.024	1			
	PSP	2.0*10^−6^	0.022	1		
	AD	0.418	0.167	3.4*10^−4^	1	
	N	0.243	0.006	9.7*10^−6^	0.123	1

*PD* Parkinson’s disease, *AD* Alzheimer’s disease, *DLB* Dementia with Lewy bodies, *PSP* Progressive supranuclear palsy, *N* Normal control.

**Table 3:** Results of ROC analysis for specificity, sensitivity and area under the curve (AUC) for aSyn and Tau aggregates in CSF.

		Specificity	Sensitivity	AUC
aSyn	PD vs. N	73.3%	64.3%	0.678
	DLB vs. N	60.0%	84.2%	0.705
	(PD + DLB) vs. N	73.3%	64.9%	0.682
Tau	(PSP + AD) vs. (PD + DLB + N)	67.6%	58.6%	0.649
	PSP vs. (PD + DLB + N)	85.5%	70.0%	0.753
	PSP vs. (AD + PD + DLB + N)	87.0%	70.0%	0.755
	AD vs. (PD + DLB + N)	19.6%	96.4%	0.462

*PD* Parkinson’s disease, *AD* Alzheimer’s disease, *DLB* Dementia with Lewy bodies, *PSP* Progressive supranuclear palsy, *N* Normal control, *AUC* Area under the curve.

The concentrations of Tau aggregates in PSP samples were significantly elevated compared to all other groups (PD, DLB, AD and controls; Fig. 9b). ROC analysis (Fig. 9d) for this model showed a sensitivity and specificity of 87 and 70% (AUC 0.76) for distinguishing PSP from all other subjects based on Tau sFIDA alone. Moreover, patients with DLB had elevated levels of Tau aggregates compared to the control group (*p* = 0.006) and to PD patients (*p* = 0.024). Interestingly, no significant increase in Tau aggregate concentration for AD patients was observed. The performance of ROC analysis for the tauopathies PSP and AD versus non-tauopathies (DLB, PD, N) revealed a decreased sensitivity and specificity compared to PSP alone. AD alone showed no distinguishability to PD, DLB, and N (Table 2).

### Aggregate concentrations show comparable discrimination to conventional biomarkers

**Fig. 10:**
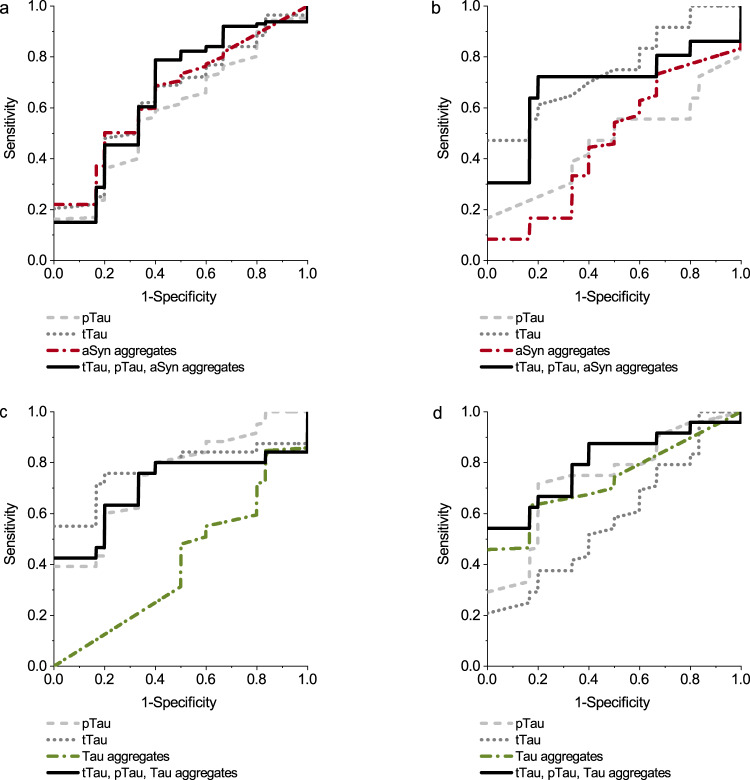
ROC of pTau, tTau, aSyn and Tau aggregates and their combination. We compared the performance of different biomarkers and analyzed, if the combination of biomarkers improves the discrimination of neurodegenerative diseases compared to normal control (**a**: PD vs. N, **b**: DLB vs. N, **c**: AD vs. N, **d**: PSP vs. N). Sensitivity, Specificity and AUC values are listed in Table 4.

In CNS biomarker research and clinical routine, total Tau protein (tTau) and phosphorylated Tau protein (pTau) are frequently used as a measure of neurodegeneration. For the present study, we received pTau and tTau concentrations of 88% of the CSF samples and compared sensitivity, specificity and AUC values for each biomarker alone and as combination of three biomarkers (for PD and DLB: pTau, tTau and aSyn aggregates, for AD and PSP: pTau, tTau and Tau aggregates). For PD vs. N, tTau, aSyn aggregates and the combination of pTau, tTau and aSyn aggregates showed nearly the same AUC but differences in specificity and sensitivity (Fig. 10 and Table 4). Due to the reduced number of samples and adaption of the method for the analysis of DLB vs. N, the AUC for aSyn aggregates was decreased compared to the first analysis with all samples (Fig. 9 and Table 3). Consequently, in this analysis, discrimination is only possible based on tTau values. Like in the first analysis, Tau aggregate levels did not discriminate AD vs. N, while AD patients showed increased concentrations of pTau and tTau and can be discriminated with an AUC of 0.78 and 0.75, respectively. For PSP vs. N, pTau and Tau aggregates separated the diseases with an AUC of 0.74 and 0.73. Here, the combination of the three biomarkers showed the largest AUC of 0.80.

**Table 4:** Results of ROC analysis for specificity, sensitivity and area under the curve (AUC) for pTau, tTau, aSyn or Tau aggregates in CSF and their combination.

		Specificity	Sensitivity	AUC
PD vs. N	tTau	60.0%	68.5%	0.645
	pTau	66.6%	55.0%	0.589
	aSyn aggregates	80.0%	50.2%	0.656
	pTau + tTau + aSyn aggregates	60.0%	78.8%	0.663
DLB vs. N	tTau	100.0%	47.2%	0.752
	pTau	100.0%	16.7%	0.468
	aSyn aggregates	100.0%	8.3%	0.468
	pTau + tTau + aSyn aggregates	80.0%	72.2%	0.689
AD vs. N	tTau	80.0%	75.8%	0.775
	pTau	66.6%	74.7%	0.753
	Tau aggregates	16.6%	84.7%	0.409
	pTau + tTau + Tau aggregates	80.0%	63.3%	0.708
PSP vs. N	tTau	100.0%	20.8%	0.586
	pTau	80.0%	71.7%	0.743
	Tau aggregates	83.3%	63.2%	0.734
	pTau + tTau + Tau aggregates	100.0%	54.2%	0.800

### aSyn and Tau aggregate concentrations significantly correlate between all patient cohorts

**Fig. 11:**
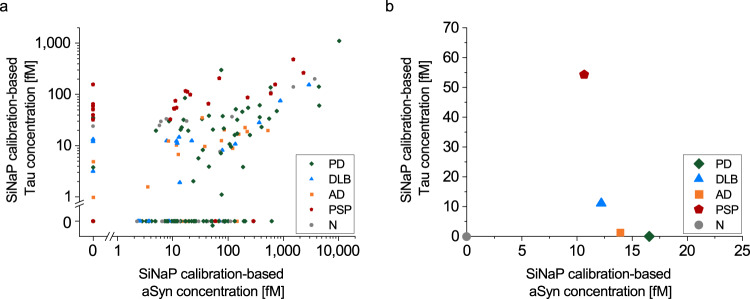
Correlation of aSyn and Tau aggregate concentration. **a** aSyn and Tau aggregate concentrations measured by sFIDA show a highly significant correlation across all samples tested (Pearson coefficient of correlation *r* = 0.81, *p* = 3.8*10^−57^) as well as for each individual cohort. Correlation of the median values for the disease groups is plotted in **b**.

As a correlation between total aSyn (t-aSyn) and total Tau (tTau) has been reported in many studies^8,22–24^, we investigated the correlation between aggregated forms of aSyn and Tau, respectively. A significant correlation between Tau and aSyn aggregate concentrations was observed for the whole data set (Pearson coefficient of correlation: *r* = 0.81, *p* = 3.8*10^−57^), as well as for each individual diagnostic group (Fig. 11a). The greatest correlation was observed for DLB samples with a Pearson coefficient of correlation of 0.98 (*p* = 8.5*10^−13^). PD (*r* = 0.87, *p* = 6.5*10^−36^), PSP (*r* = 0.74, *p* = 3.4*10^−6^), and normal control (*r* = 0.90, *p* = 2.5*10^−17^) CSF samples also showed a positive correlation. For AD patients, the correlation was weaker (*r* = 0.52, *p* = 0.005).

### Age, sex and disease duration do not correlate with aggregate concentrations in CSF

As age and gender are risk factors for PD, AD, DLB and PSP^25^, the correlation between the concentration of aSyn and Tau aggregates to age and sex are interesting parameters. Across all cohorts, there was no detectable significant effect specific to age, sex, or disease duration (Pearson coefficient of correlation in Supplementary Table 6). For Tau, we observed an inverse correlation between aggregate concentration and education.

## Discussion

Our study explored the ability of the sFIDA technology to detect and quantitate aSyn and Tau aggregates in CSF samples and its applicability for the diagnosis of neurodegenerative diseases. aSyn oligomers are thought to be the major toxic species in synucleinopathies like PD and DLB^4,26^ but the detection of such oligomers in human biofluids is still challenging due to the low concentration of oligomers and the interference with monomers. The principle of sFIDA allows the sensitive detection and quantitation of oligomers and other aggregates in the presence of monomers with an LOD in the low femtomolar region. Approximately 66% of the CSF samples tested here showed concentrations above the LOD with a wide concentration range of up to 10 pM. Most samples harbored concentrations of aSyn aggregates between 5 fM and 500 fM. For Tau aggregates, 44% of the CSF samples were above the LOD. It has to be considered that a single SiNaP led on average to more pixels with fluorescence above the cutoff value than compared to the average aggregate from real samples. This is presumably due to a higher amount of accessible binding sites for detection antibodies or due to agglomeration of our silica nanoparticle standard. Probably, both aspects influence the average apparent size distribution of our standard particles. With the term size, we therefore do not refer to the actual size, as all particles can be expected to be below the optical resolution limit, but instead to the number of pixels that are illuminated above the cutoff value. The evaluation accounts at least partially for that, because the exact fluorescence intensity of a pixel is not affecting the readout, only the digital decision, whether the fluorescence intensity of a pixel is above the cutoff threshold or not. Nevertheless, the differences in particle size may influence the calibration, so we described the calibrated concentrations as *SiNaP calibration-based concentration*. In this study, we calculated an average apparent particle size of 11.2 pixels per particle for aSyn SiNaPs (obtained from the 63 fM calibration), while for aggregated aSyn in patient samples, the apparent average particle size was 5.2 pixels. For Tau, 204 fM SiNaPs and samples yielded an average apparent particle size of 10 pixels and 2.6 pixels, respectively.

As expected, CSF samples of PD and DLB patients harbored significantly elevated levels of aSyn aggregates compared to normal controls. This is in agreement with several other studies quantifying aSyn oligomers in CSF^8,27,28^. But there is also a large overlap between synucleinopathies and normal controls, which is congruent with some previous studies aimed at discriminating both populations (Majbour: sensitivity 89%, specificity 52%^8^, Tokuda: sensitivity 75%, specificity 88%^27^). The combination of aSyn aggregates with other predictive values like total aSyn (t-aSyn), phosphorylated aSyn (p-aSyn), tTau, phosphorylated Tau (pTau), or age may improve the discrimination of synucleinopathies from normal controls, as investigated in other studies^8,21,29^. In this work, we have tested the combination of aSyn aggregates as biomarker with pTau and tTau. The combination of the three biomarkers did not improve the predictive power of the analysis, but for PD vs N, aSyn aggregates alone showed the highest performance of the three biomarkers. We hypothesize that the combination with t-aSyn probably has a higher impact on the AUC, but information on total aSyn levels was not available for the samples tested in this study.

Interestingly, we found elevated levels of aSyn aggregates also in AD patients with concentrations comparable to that of PD or DLB patients. The role of aSyn in AD is still under investigation. Many studies have reported the presence of Lewy bodies in AD brains^30–33^ as well as increased t-aSyn concentrations in CSF^24^, and in an autopsy study based at UPenn, where our CSF samples were collected, more than 52% of individuals with a diagnosis of AD showed considerable Lewy body burden on neuropathology^34^. However, prior biomarker studies have also reported no difference in aSyn monomer concentrations in CSF of AD patients compared to normal controls^21^, or even decreased levels of aSyn oligomers in CSF of AD and PSP compared to PD patients^27,35^, which is in contrast to our results. Differences in study results might be ascribed to differences in (1) the makeup of patients recruited at different clinical sites, (2) preanalytical aspects related to sample collection or handling, or (3) quantification methods^36^. In this context, we note that all samples used in this study were single-use aliquots collected under strict standard operating procedures. For PSP, we did not measure a significant increase in aSyn aggregate concentration compared to normal controls, which agrees with other studies^21,27^.

To date, limited evidence exists regarding the detectability of Tau aggregates in CSF for the diagnosis of neurodegenerative diseases. Increased Tau oligomer concentrations in postmortem PSP brain samples have been reported by Gerson et al.^37^. This is in agreement with our study, where PSP patients showed increased levels of Tau aggregates compared to all other diseases, with sensitivity and specificity of 87 and 70%, respectively. Although there is a consensus about the certainty of tTau and pTau for diagnosis of AD^38^ and the presence of Tau oligomers in AD brains^9^, we did not observe a statistically significant increase in the concentration of Tau aggregates in CSF samples of these patients. Up to now, most studies are focusing on the presence of tTau and pTau in neurodegenerative diseases. For PSP and AD, quite different concentrations are found in CSF: AD shows significantly increased levels of tTau and pTau^23^, whereas PSP samples show no difference or even a decrease in Tau monomer concentrations^23,39^. These observations match our data showing that pTau and tTau were increased for AD, but decreased for PSP (for pTau). For PSP, Tau aggregates alone can differentiate between PSP and normal control group similar to pTau, and the combination of the biomarkers improves the specificity and AUC, which underlines the role of Tau aggregates in PSP as possible biomarker. Wagshal et al. postulate that differences between AD and PSP can probably be ascribed to differences in Tau isoforms, as PSP is known as a 4R-tauopathy, whereas AD shows equal ratios of 4 R and 3 R Tau. Different isoforms of Tau are differentially released from neuronal and glial cells and have differing affinities to antibodies^39,40^. These differences could also be relevant in interpreting our present results, which suggest that aggregated Tau species discriminate PSP vs. AD.

The importance of Tau protein in PD and DLB is still under investigation. Many studies have reported the presence of neurofibrillary tangles in PD and DLB brains^30,31,41,42^ but no increase in tTau or pTau in CSF samples of PD patients^8,21^. Our study implicates no relation of increased Tau aggregate concentrations in CSF and the presence of PD. Interestingly, DLB samples showed elevated levels of Tau aggregates compared to those of normal controls and compared to PD patients, but less than those observed in PSP samples.

We also correlated aSyn and Tau aggregate concentrations in CSF samples between individual groups. For correlation of t-aSyn and tTau evidence in the literature is inconcise. Parnetti et al. reported an inverse correlation of aSyn and Tau^29^, while several others have observed a positive correlation^22–24^. For aSyn and Tau aggregates, we observed a highly significant positive correlation (Fig. 11a), which is in agreement with several other studies showing the coexistence of the two proteins in Lewy bodies^30,41^ and even the existence of hetero-aggregates^33^. Despite substantial overlap, median values of the individual disease groups suggest a mixed pathology ranging from rather pure aSyn pathology in PD via AD and DLB to PSP which shows decreased aSyn and increased Tau pathology (Fig. 11b). Additional correlations with other potential biomarkers, e.g. Amyloid beta and TDP43 aggregates, may further complement the view on these diseases on the molecular level. Determining whether aSyn-Tau hetero-aggregates might be detected by sFIDA in human biofluids is a promising area of future investigation that might add to our understanding on the molecular basis of phenotypic overlap among neurodegenerative diseases.

Naturally occurring oligomers and other aggregates differ in size, morphology and posttranscriptional modifications^4,43^. For detection and quantification, we used the same capture and detection antibody directed against linear epitopes that are expected to be accessible in all aggregated species, in order to quantitate all isoforms, irrespectively of their structural conformation. In future studies, we will further characterize the exact nature of the analytes by introducing size standards and structural probes. Possibly, not all aSyn assemblies in human brain are neurotoxic or disease-specific, and it was strongly discussed if aSyn physiologically occurs as a globular tetramer or as an intrinsically disordered monomer^4,44,45^. Future research will show, if the complex pathology of neurodegenerative diseases limits the diagnostic specificity of measuring the whole soluble aggregate fraction. Nevertheless, we are convinced that the possibility to finally measure aggregate concentrations is essential not only in understanding the underlying pathology, but also for developing therapeutic compounds against these species. Here we showed, that total aggregate concentrations differentiate i.e. PD or PSP from normal control, which further emphasizes the usefulness of quantifying aggregates in CSF for diagnosis of neurodegenerative diseases. For sufficient accuracy, surely, sensitivity and specificity need to be improved, i.e., by combining the concentration of aSyn or Tau aggregates with other biomarkers like t-aSyn, tTau, pTau, or Aβ1-42. Taking further into consideration that perhaps not all naturally occurring aggregates are disease-relevant, it might be interesting to compare or combine the results of total aggregates measured by sFIDA with seeding assays like the RT-QuIC or assays that measure a specific fraction of aggregates.

Moreover, we note that, aside from diagnostic applications, sFIDA may be a valuable tool in clinical studies, to select, stratify, and monitor patients for therapies targeting aSyn or Tau oligomers, since sFIDA allows for direct assessment of the mechanism of action and is able to measure target engagement, irrespectively of the structural conformation. Finally, treatment success can be validated on the molecular level by monitoring aggregate titers over the course of medication.

## Methods

### Synthesis of protein-coated silica nanoparticles

For assay calibration we have developed a nanoparticle calibration standard based on a silica core^14^. These silica nanoparticles (SiNaPs) were synthesized via Stöber process and afterwards modified with 3-aminopropyl(triethoxysilane) (APTES, Sigma-Aldrich, St. Louis, USA) to generate an aminated surface. Proteins were crosslinked to the aminated surface by maleimido hexanoic acid (MIHA, abcr GmbH, Karlsruhe, Germany). After activation with 200 mM 1-ethyl-3-(3-dimethylaminopropyl)carbodiimide (EDC, Sigma Aldrich, St. Louis, USA) and 50 mM N-hydroxysuccinimid (NHS, Sigma Aldrich, St. Louis, USA) for 10 min at room temperature (RT), the carboxy group of MIHA was coupled covalently to the amines of the silica nanoparticles. Following incubation for 1 h at RT, the resulting SiNaPs were centrifuged (7000 x *g*, 2 min) and redispersed in PBS and 10% dimethylformamid (DMF, Sigma Aldrich, St. Louis, USA). The washing step was repeated three times, where after the pellet was redispersed in PBS containing 10% DMF and 50 mM ethylendiamintetraacetic acid disodium salt (Na_2_EDTA, AppliChem, Darmstadt, Germany) in the last step. Protein fragments of aSyn (aa115-130, Peptides and Elephants, Henningsdorf, Germany) and Tau (aa 210-230, Peptides and Elephants, Henningsdorf, Germany) are functionalized with cysteamine on the C-terminus to enable reacting with the maleimide group of the SiNaPs. For synthesis of protein-conjugated silica nanoparticles, 10% of the possible binding sites were functionalized by adding protein to the redispersed SiNaPs. The dispersion was shaken at RT and 650 rpm. After 1 h, 50 µL of 1 M Tris-(2-carboxyethyl)-phosphine (TCEP, abcr GmbH, Karlsruhe, Germany) was added to prevent oxidation of the protein. The reaction was quenched by adding 20 µL of a 1 M 2-mercaptoethanol solution. The functionalized SiNaPs were washed two times by centrifugation (10,000 x *g*, 4 min) and redispersed in ddH_2_O. Finally, the silicon concentration was determined using ICP-MS (inductively coupled plasma – mass spectrometry) and the resulting molar SiNaPs concentration was calculated based on size, density as well as particle shape. Prior to use, the protein-conjugated silica nanoparticles were subjected to ultra-sonification for 10 min.

Tau and aSyn monomers were isolated prior to sFIDA measurement using size exclusion chromatography (Bio SEC3, pore size 150 Å, Agilent, Santa Clara, USA) to ensure that the sample does not contain any aggregates. After SEC purification, we determined the monomer concentration using UV-Vis spectroscopy. We calculated the signal reduction of monomers versus aggregates as described in Eq. (1):

1 $$Signal\,reduction\left[ {{{\mathrm{\% }}}} \right] = \left( {1 - \frac{{pixel\,count_{monomer} - pixel\,count_{BC}}}{{pixel\,count_{aggregates} - pixel\,count_{BC}}}} \right)\ast 100{{{\mathrm{\% }}}}$$

### Characterization of silica nanoparticles

Size and particle shape of the aminated silica nanoparticles were analyzed using transmission electron microscopy (TEM) as previously described by Hülsemann et al.^14^. Mean particle size was 18.5 nm for the aminated silica core (TEM image and size distribution in Supplementary Fig. 1).

Finally, the silicon concentration was determined using inductively coupled plasma – mass spectrometry (ICP-MS). SiNaPs were diluted in 3% nitric acid and analyzed in helium collision cell mode with an Agilent 7500 (Agilent Technologies, Japan). External calibration with rhodium as the internal standard was performed using NIST traceable commercial standard solution (VWR International, PA, USA). Complete dissociation of silica nanoparticles in the plasma without the need for digestion prior to analysis was shown in earlier studies up to a particle diameter of 500 nm^46–48^. The molar SiNaPs concentration was calculated based on the silicon concentration determined by ICP-MS and the known size, density as well as shape of the particles.

### Labeling of antibodies

For microscopic detection of aggregates, we used fluorescent antibodies. The mouse anti-aSyn monoclonal antibody 211 (Santa Cruz Biotechnology, Inc., Dallas, USA) was labeled with CF633 (Biotium, Freemont, USA), whereas the anti-tau Tau5 antibody (Biolegend, San Diego, USA) was labeled with CF488A (Biotium, Freemont, USA). The labeling process was performed as described in the manufacturer’s protocol. The dyes were activated as succinimidyl esters to react covalently with the amines of the antibody in carbonate buffer. For purification of each labeled antibody, a polyacrylamide bead suspension (Bio-Gel P-30 Gel, Bio-Rad Laboratories, Inc., Hercules, USA) was used. The concentration and the degree of labeling was determined according to the manufacturer’s protocol.

### Assay protocol

The biochemical principle of the sFIDA assay was previously described by Kravchenko et al., and Herrmann et al.^11,49^. In the present study, we used Nunc MicroWell 384-Well plates (Thermo Fisher Scientific, Waltham, USA) functionalized with 211 and Tau5 antibodies as captures, each at 5 µg/mL in 1 x PBS buffer. After washing five times with 80 µL TBS-T (1x TBS (Serva, Duisburg, Germany) and 0.05% Tween20 (AppliChem, Darmstadt, Germany)) and afterwards five times with 1 x TBS, the wells were blocked with 1% BSA (AppliChem, Darmstadt, Germany) in TBS containing 0.03% ProClin (Sigma Aldrich, Missouri, USA) for 1.5 h at RT. The plate was washed again with TBS-T and TBS (see above) and 20 µl protein-conjugated SiNaPs diluted in TBS-ProClin containing 0.5% BSA and 0.05% Tween, and 20 µl of the samples were incubated for 2 h at RT. After washing five times with TBS and changing the buffer to TBS-ProClin, the wells were incubated for 2 h with the fluorescent detection antibodies 211-CF633 (0.4 µg/mL) and Tau5-CF488 (4 µg/mL) in TBS, after which the wells were washed with TBS again. For measurement, the buffer in the wells was changed against TBS-ProClin. Each concentration and sample were pipetted fourfold. All washing steps were carried out by an automated microplate washer (405 LS Microplate Washer, BioTek, VT, USA).

### Inter-assay and inter-laboratory measurements

For inter-assay measurement of the calibration curve and the samples, the same assay was repeated four months later by the same technician with the same antibodies and materials but minor changes in washing conditions, such as the use of a different microplate washer and washing and blocking reagents with a different manufacturing date. Repeatedly assayed samples were subjected to an additional freeze-thaw cycle.

For inter-laboratory analysis, the assay was prepared and measured by a different operator in a different laboratory. The first measurement took place at the Forschungszentrum Jülich, and the second measurement at the Heinrich-Heine-Universität in Düsseldorf two months after the first measurement with the same changes as described above for inter-assay analysis.

### Immunodepletion

For immunodepletion, 211 and Tau5 antibody were covalently coated to carboxylated magnetic dynabeads (Invitrogen, Waltham, USA) according to the manufacturer’s protocol. Shortly, dynabeads were washed twice with 2.5 mM 2-(N-morpholino)ethanesulfonic acid (MES, pH 5, Roth, Karlsruhe, Germany) and applied to a magnet to remove the supernatant. Carboxy groups were activated with 50 µg/ml EDC and 50 µg/ml NHS in MES for 30 min at RT while rotating. After activation, the dynabeads were washed again with MES and coated with 211 or Tau5 antibody to a concentration of 20 µg/ml dynabeads, respectively. To ensure that signal loss is not due to unspecific binding of sample components to dynabeads, we run a third synthesis without antibody. After incubation for 1 h at RT, dynabeads were washed again and quenched with 50 mM ethanolamine in MES for 1 h at RT followed by a last washing step.

For immunodepletion, we applied 0.5 mg of antibody coated dynabeads to the magnet and removed the supernatant. 100 µl sample were added and incubated for 1 h at RT while rotating. After incubation, dynabeads were applied to the magnet again and the supernatant was transferred to a fresh tube. The immunodepleted samples were analyzed using sFIDA as described above. To consider for possible effects of magnetic beads on the pixel count, we normalized the signals by using an individual cutoff based on the CSF control. Please, note that the CSF control used for immunodepletion and HAMA interference experiments differed from the CSF control used for calibrating the results of the big data set of the study and showed an increased fluorescence signal for Tau5 CF488.

### Influence of heterophilic antibodies

The potential influence of heterophilic antibodies, specifically anti-mouse antibodies (HAMAs), was analyzed using the purified mouse IgG isotype control MOPC-21 as a competitor (Biolegend, San Diego, USA). Possibly existing HAMAs in CSF can bind to MOPC instead to the assay antibodies which prevents false positive signals. A total of nine samples that yielded high sFIDA signals were spiked with 1 µg/ml MOPC-21. As positive control, we used buffer spiked with 1 µg/ml goat anti-mouse IgG (Thermo Fisher Scientific, Waltham, USA) with or without MOPC-21.

### Determination of blood contamination

Contamination of CSF samples with blood was determined semi-quantitatively using Combur10-Test-Analysis (Hoffmann-La Roche, Basel, Switzerland) as described in Barkovits et al.^20^. Test stripes were incubated with 50 µL CSF for 60 s and the amount of contamination was analyzed according to the manufacture’s protocol.

### Image-data acquisition

Imaging was performed on a total internal reflection microscope (TIRFM, Leica DMI6000B, Wetzlar, Germany) as previously described by Kravchenko et al.^49^ (excitation: 635 nm, emission filter: 705/22 nm; excitation: 488 nm, emission filter: 525/36 nm; exposure time: 1000 ms; gain: 1300). A total of 25 images per well with 1000 × 1000 pixels each were measured, which covers 3.14% of the total area per well. For unbiased and automated image-data analysis, we have used our previously developed sFIDAta software tool^15^. The analysis includes the automated detection and elimination of artefact containing images and counting of aggregate indicating pixels. The *pixel count* is referred to as the average number of pixels in an image that exceed a pre-defined cutoff value. The *cutoff* is defined as the grey-scale value at which the ratio of the positive versus the total number of pixels in the buffer control equals a pre-defined value. The cutoff is used to compensate fluctuations in the absolute fluorescence intensities among experiments and different conditions within one experiment (i.e., antibody dilutions) and is determined for each experiment based on a buffer control sample. To further ensure that differences in fluorescence intensity do not affect assay robustness, we run a calibration in each 384-microtiter plate and converted pixel counts into SiNaP calibration-based fM concentrations.

For inter-assay measurement, a cutoff of 0.001% was chosen, while the cutoff for the analysis of the whole dataset of 237 samples was 0.05%. This difference is due to a new lot of detection antibodies with a lower degree of labeling. To further ensure that all artificial images are excluded from the analysis, min-max filtering was applied, which removed 10% of the images per well with the highest and 10% of the images per well with the lowest pixel counts.

### Statistics

#### General statistics

Statistical analysis was performed using OriginPro 2020 SR1 (OriginLab Corporation, MA, USA) and matlab2019b (The MathWorks, MA, USA) software. Mean and standard deviation was calculated based on the pixel counts of the four replicates. Intra-assay variation is described by the CV% value. To determine inter-assay and inter-laboratory variation, the Pearson coefficient of correlation was calculated for the replicate measurements of the samples.

#### Calibration

For calculation of the calibration curve, only the concentrations of the silica nanoparticle standard were included that significantly differed from the blank control and were above the limit of detection (LOD). To this end, a one-sided Mann-Whitney U test was carried out with a confidence interval of 5%. After calculation of the calibration range for each experiment, a universal calibration range for all of the experiments was established. The LOD is defined based on Eq. (2):

2 $$LOD\left[ {pixel} \right] = pixel\,count(blank\,control)\ast 2\sigma$$

For linear regression, the pixel counts were weighted with 1/readout. The bovine CSF control was used as a negative control for the calibration as well as for calculation of the LOD.

#### Logistic regression and ROC analysis

Logistic regression was performed to evaluate the ability of each biomarker to classify the diagnostic groups. To this end, we used scikit-learn library (version 1.0.2). Since the use of multiple features increases the risk of overfitting, the k-fold cross-validation method was used to generate Fig. 10, in order to provide unbiased results. Deviations between Fig. 9 and Fig. 10 can be explained not only by the modified method but also by a divergent data basis. Since tTau and pTau values were not available for all samples, these were excluded for the creation of the Fig. 10. By forming the average of the k results, a single receiver operating characteristic (ROC) curve was generated. The optimal combination of sensitivity and specificity for a ROC curve was calculated with a maximized Youden’s index.

### Patient samples

Patients were recruited from the University of Pennsylvania (UPenn) Parkinson’s Disease and Movement Disorder Center (PDMDC), Alzheimer’s Disease Center (ADC), or Frontotemporal Dementia Center (FTDC). Written, informed consent was obtained from each study participant at enrollment and biofluids were collected and stored for future research as approved by the UPenn Institutional Review Board (FWA00004028). Participants were diagnosed with Parkinson’s disease (PD, *n* = 115), Alzheimer’s disease (AD, *n* = 28), progressive supranuclear palsy (PSP, *n* = 30), or dementia with Lewy bodies (DLB, *n* = 19) according to clinical criteria as previously described^50^. Participants with no known neurological disorder were also enrolled (normal control, N, *n* = 45). Cerebrospinal fluid (CSF) was collected by trained neurologists via lumbar puncture, and aliquots of 0.5 mL were stored at −80 °C until analysis. Demographic information was collected by trained research staff. Samples were collected between August of 2005 and November 2019, with the exception of one sample, which was collected in March of 1999. Samples included in the analysis were matched for age across diagnosis groups. Initially, a subset of PSP (*n* = 30), PD (*n* = 30), and N (*n* = 30) samples were analyzed as an exploratory cohort. The remainder of the samples were analyzed to investigate differences between disease groups. Researchers were blinded to clinical data at the time of sFIDA measurement.

Concentrations of pTau and tTau were measured using Luminex xMAP immunoassay platform (Luminex, Austin, USA)^51,52^ and provided by Integrated Neurodegenerative Disease Database (INDD).

After unblinding, the data points of each group were first tested for normal distribution (Shapiro Wilk, Lilliefors, Kolmogorov-Smirnov, Anderson Darling). Afterwards, a Kruskal-Wallis test was executed to identify differences between the groups. In case of significant differences (*p* < 0.05) a pairwise comparison using the two-sided Mann-Whitney U test with a confidence interval of 0.05 was performed.

## Supplementary information

Supplementary Information

## Data Availability

The authors confirm that the data supporting the findings of this study are available within the article and its supplementary materials.

## Code availability

For image data analysis, we used the sFIDAta software tool, which can be made available upon request from the corresponding author.
