# Supplementary material for: Quantitative detection of α-Synuclein and Tau oligomers and other aggregates by digital single particle counting
Source: NPJ Parkinsons Dis. 2022 Jun 2;8:68. doi: 10.1038/s41531-022-00330-x (PMC9163356; doi:10.1038/s41531-022-00330-x)
Supplement: Supplementary file 1 — Supplementary Information [file 41531_2022_330_MOESM1_ESM.pdf]

Quantitative Detection of α-Synuclein and Tau Oligomers and other Aggregates by Digital Single Particle Counting

Lara Blömeke, Marlene Pils, Victoria Kraemer-Schulien, Alexandra Dybala, Anja Schaffrath, Andreas Kulawik, Fabian Rehn, Anne Cousin, Volker Nischwitz, Johannes Willbold, Rebecca Zack, Thomas F. Tropea, Tuyen Bujnicki, Gültekin Tamgüney, Daniel Weintraub, David Irwin, Murray Grossman, David A. Wolk, John Q. Trojanowski, Oliver Bannach, Alice Chen-Plotkin, Dieter Willbold

Supplementary Information

Supplementary Table 1 CV% values of each experiment for SiNaPs and CSF samples

	aSyn		Tau	
Experiment	Calibration	Samples	Calibration	Samples
1	18.05	22.08	20.42	18.62
2	22.18	32.86	30.27	19.45
3	17.33	20.71	23.00	15.66
4	14.63	12.58	18.60	10.81
5	11.71	9.16	12.54	8.66
6	14.87	12.70	16.01	9.58
7	16.99	13.86	15.15	10.15
8	10.29	10.58	16.77	10.84
Mean	15.76	16.82	19.10	12.97

Supplementary Table 2 LOD values for aSyn- and Tau silica nanoparticles [fM] for each experiment

Experiment	aSyn	Tau
1	9.59	36.69
2	20.72	70.09
3	6.76	32.04
4	1.67	10.09
5	6.08	-
6	2.43	-
7	3.53	-
8	2.98	19.41
Mean	6.72	33.7

Supplementary Table 3 Number of samples above the LOD for each individual experiment

Experiment	aSyn	Tau
1	11	8
2	13	6
3	18	19
4	23	9
5	17	5
6	27	24
7	20	15
8	27	18
Sum	156	104

Supplementary Table 4 Demographic information, aSyn and Tau aggregate concentrations and blood contamination level of each individual patient CSF sample

Sample	*Diagnosis*	*Sex*	*Deceased*	*Education (years)*	*Age at Sample*	*aSyn aggregate concentration [fM]*	*StdDev aSyn*	*Tau aggregate concentration [fM]*	*StdDev Tau*	*Blood contamination level*
1	AD	Male	True	16	72.00	0.00	0.00	0.00	0.00	0
2	AD	Female	True	12	68.00	3.51	0.30	1.60	0.23	0
3	AD	Male	False	18	75.00	8.84	0.36	0.00	0.00	0
4	AD	Male	True	18	75.00	24.20	3.20	0.00	0.00	0
5	AD	Female	False	6	65.00	75.75	12.31	12.70	0.38	0
6	AD	Female	True	15	65.00	0.00	0.00	0.00	0.00	0
7	AD	Female	False	18	65.00	521.47	116.08	20.11	2.04	4
8	AD	Male	False	11	77.00	9.52	1.14	0.00	0.00	0
9	AD	Female	False	16	76.00	18.48	1.37	0.00	0.00	4
10	AD	Male	True	16	61.00	144.55	14.95	0.00	0.00	0
11	AD	Male	True	12	68.00	0.00	0.00	0.00	0.00	0
12	AD	Male	False	18	66.00	66.46	7.84	0.00	0.00	0
13	AD	Male	False	20	81.00	202.30	16.43	22.69	2.87	0
14	AD	Female	False	16	75.00	156.65	32.57	16.87	2.12	0
15	AD	Male	True	12	69.00	15.41	1.25	0.00	0.00	2
16	AD	Female	False	18	70.00	0.00	0.00	0.00	0.00	1
17	AD	Female	False	20	75.00	0.00	0.00	0.00	0.00	0
18	AD	Male	False	12	64.00	0.00	0.00	0.00	0.00	3
19	AD	Male	False	12	73.00	221.12	31.78	19.19	1.78	4
20	AD	Male	False	12	63.00	12.42	2.29	6.79	0.53	4
21	AD	Male	True	16	66.00	37.81	6.06	9.82	0.83	0
22	AD	Male	False	18	68.00	33.69	2.30	35.38	3.16	1
23	AD	Female	False	15	69.00	0.00	0.00	4.93	0.50	4
24	AD	Male	False	12	56.00	0.00	0.00	0.99	0.06	1
25	AD	Male	True	14	55.00	11.58	1.01	10.33	1.09	0
26	AD	Female	False	12	68.00	120.74	16.88	8.98	0.83	0
27	AD	Male	False	16	66.00	8.32	0.66	12.36	0.66	2
28	AD	Male	False	16	59.00	85.40	9.02	20.72	1.71	4

29	DLB	Male	True	12	73.00	12.20	1.76	11.07	1.02	0
30	DLB	Male	False	12	67.00	8.52	1.31	0.00	0.00	0
31	DLB	Female	False	18	68.00	2.57	0.36	0.00	0.00	3
32	DLB	Male	False	16	79.00	886.05	65.57	75.02	4.34	0
33	DLB	Female	False	20	58.00	0.00	0.00	12.05	0.74	0
34	DLB	Female	True	12	66.00	13.02	1.20	14.56	2.10	0
35	DLB	Female	False	14	87.00	2.38	0.22	0.00	0.00	2
36	DLB	Male	False	18	66.00	70.49	6.04	0.00	0.00	0
37	DLB	Male	True	20	66.00	3.75	0.15	0.00	0.00	0
38	DLB	Male	False	14	74.00	0.00	0.00	3.14	0.51	2
39	DLB	Male	True	19	65.00	7.88	0.84	12.38	0.96	0
40	DLB	Male	True	12	70.00	0.00	0.00	13.39	0.53	0
41	DLB	Male	False	12	72.00	370.27	51.32	27.96	3.86	0
42	DLB	Male	False	16	68.00	22.03	3.69	12.43	0.76	0
43	DLB	Male	True	18	63.00	78.99	3.33	8.12	0.60	2
44	DLB	Male	False	14	84.00	13.47	1.75	1.89	0.14	1
45	DLB	Male	False	16	68.00	2949.10	470.68	153.28	17.66	0
46	DLB	Male	False	16	63.00	136.01	24.98	10.69	1.15	2
47	DLB	Female	False	16	68.00	11.73	1.42	12.32	0.51	0
48	N	Female	False	18	70.00	0.00	0.00	0.00	0.00	3
49	N	Female	False	18	73.00	81.98	22.45	0.00	0.00	0
50	N	Male	False	16	57.00	0.00	0.00	0.00	0.00	0
51	N	Male	False	12	61.00	0.00	0.00	36.89	7.70	2
52	N	Male	False	18	74.00	0.00	0.00	0.00	0.00	0
53	N	Female	True	20	70.00	0.00	0.00	0.00	0.00	0
54	N	Male	True	19	83.00	0.00	0.00	0.00	0.00	4
55	N	Female	False	18	74.00	0.00	0.00	24.30	4.41	1
56	N	Male	False	18	75.00	0.00	0.00	0.00	0.00	3
57	N	Female	False	16	58.00	43.36	8.97	0.00	0.00	0
58	N	Male	False	16	67.00	119.16	46.24	37.25	5.88	1
59	N	Female	False	20	69.00	0.00	0.00	0.00	0.00	3
60	N	Male	False	20	60.00	72.13	8.67	7.79	0.62	0
61	N	Male	False	12	62.00	0.00	0.00	0.00	0.00	0
62	N	Male	False	20	77.00	0.00	0.00	0.00	0.00	0

63	N	Female	False	18	63.00	0.00	0.00	0.00	0.00	0
64	N	Female	False	12	59.00	602.80	231.60	110.63	16.15	3
65	N	Female	False	20	57.00	13.13	2.78	0.00	0.00	1
66	N	Male	True	12	93.00	0.00	0.00	0.00	0.00	4
67	N	Female	False	18	80.00	0.00	0.00	0.00	0.00	2
68	N	Male	False	20	70.00	5.00	0.27	0.00	0.00	0
69	N	Male	False	20	71.00	0.00	0.00	0.00	0.00	1
70	N	Female	False	20	71.00	17.92	1.72	30.74	1.94	2
71	N	Female	False	20	71.00	0.00	0.00	0.00	0.00	1
72	N	Female	True	14	89.00	0.00	0.00	0.00	0.00	0
73	N	Female	False	20	66.00	0.00	0.00	0.00	0.00	4
74	N	Female	False	16	64.00	0.00	0.00	0.00	0.00	0
75	N	Male	False	18	70.00	0.00	0.00	0.00	0.00	4
76	N	Male	True	20	90.00	112.50	45.08	0.00	0.00	4
77	N	Male	False	12	71.00	0.00	0.00	0.00	0.00	0
78	N	Male	False	4	56.00	0.00	0.00	0.00	0.00	0
79	N	Male	False	20	74.00	0.00	0.00	0.00	0.00	3
80	N	Male	False	9	66.00	0.00	0.00	0.00	0.00	0
81	N	Male	True	20	80.00	1536.16	572.99	142.57	33.72	4
82	N	Female	False	16	57.00	16.57	1.15	0.00	0.00	0
83	N	Male	False	18	70.00	0.00	0.00	0.00	0.00	4
84	N	Female	False	18	66.00	6.64	1.21	0.00	0.00	4
85	N	Male	False	18	69.00	29.53	5.15	0.00	0.00	3
86	N	Female	False	14	65.00	7.74	0.55	33.95	2.42	0
87	N	Female	False	18	64.00	5.78	0.41	25.09	2.54	3
88	N	Male	False	12	62.00	0.00	0.00	0.00	0.00	2
89	N	Female	False	14	66.00	2.25	0.23	0.00	0.00	4
90	N	Male	False	12	57.00	6.19	0.34	30.29	3.81	4
91	N	Male	False	16	74.00	3742.12	361.11	202.57	17.10	4
92	N	Male	False	18	63.00	6.47	0.46	0.00	0.00	4
93	PD	Female	False	16	63.00	0.00	0.00	34.76	12.08	0
94	PD	Male	True	16	76.00	49.27	9.72	0.00	0.00	0
95	PD	Male	True	12	75.00	10229.34	5666.03	1117.47	181.59	4

96	PD	Male	False	16	63.00	621.44	232.23	0.00	0.00	0
97	PD	Male	True	16	64.00	0.00	0.00	0.00	0.00	1
98	PD	Male	False	18	64.00	3.75	0.43	0.00	0.00	0
99	PD	Female	True	18	80.00	147.24	46.97	32.26	5.74	4
100	PD	Male	True	12	84.00	86.24	19.19	0.10	0.02	4
101	PD	Male	True	14	62.00	131.68	14.47	16.11	1.62	0
102	PD	Female	False	18	66.00	248.19	44.48	16.14	1.21	4
103	PD	Male	False	20	58.00	193.78	91.25	0.00	0.00	4
104	PD	Male	False	16	62.00	75.01	15.85	302.30	78.63	0
105	PD	Male	False	18	87.00	52.49	6.61	0.08	0.01	0
106	PD	Female	False	18	59.00	10.72	1.55	0.00	0.00	4
107	PD	Male	True	16	72.00	146.89	17.61	17.27	1.07	2
108	PD	Male	True	11	77.00	107.68	22.31	10.75	0.51	0
109	PD	Female	False	16	57.00	53.46	6.75	20.61	2.53	3
110	PD	Male	False	18	64.00	4.85	0.54	0.00	0.00	4
111	PD	Male	False	18	67.00	0.00	0.00	0.00	0.00	4
112	PD	Male	False	18	64.00	17.19	5.94	0.00	0.00	0
113	PD	Male	False	16	65.00	766.49	320.78	47.45	4.65	0
114	PD	Male	True	14	79.00	0.00	0.00	0.00	0.00	3
115	PD	Male	False	13	67.00	372.84	98.84	61.82	10.50	2
116	PD	Female	False	19	60.00	138.14	46.83	51.90	10.04	0
117	PD	Male	False	16	66.00	289.85	75.62	0.00	0.00	1
118	PD	Male	False	13	59.00	83.84	18.40	0.00	0.00	2
119	PD	Male	False	18	60.00	0.00	0.00	0.00	0.00	0
120	PD	Male	False	16	59.00	40.33	19.68	0.00	0.00	4
121	PD	Male	False	14	56.00	0.00	0.00	0.00	0.00	1
122	PD	Female	True	18	63.00	8.36	1.42	0.00	0.00	2
123	PD	Male	True	16	73.00	0.00	0.00	0.00	0.00	1
124	PD	Female	False	17	70.00	0.00	0.00	0.00	0.00	4
125	PD	Female	False	16	63.00	0.00	0.00	0.00	0.00	0
126	PD	Male	True	20	70.00	0.00	0.00	0.00	0.00	0
127	PD	Female	False	18	67.00	0.00	0.00	0.00	0.00	0
128	PD	Male	False	18	68.00	0.00	0.00	0.00	0.00	0
129	PD	Male	False	16	62.00	16.54	1.44	85.32	5.46	0

130	PD	Female	False	14	61.00	205.19	88.91	0.00	0.00	4
131	PD	Male	True	18	72.00	0.00	0.00	0.00	0.00	3
132	PD	Male	True	12	64.00	84.34	12.81	21.78	2.25	0
133	PD	Female	False	16	63.00	0.00	0.00	0.00	0.00	1
134	PD	Male	False	13	57.00	0.00	0.00	0.00	0.00	0
135	PD	Male	False	16	67.00	587.52	134.89	137.76	17.80	0
136	PD	Female	False	18	62.00	0.00	0.00	0.00	0.00	0
137	PD	Male	False	14	54.00	0.00	0.00	0.00	0.00	0
138	PD	Male	True	14	55.00	90.61	15.76	0.00	0.00	4
139	PD	Male	False	20	59.00	51.37	18.63	0.00	0.00	4
140	PD	Male	False	20	66.00	0.00	0.00	0.00	0.00	0
141	PD	Male	False	12	57.00	375.96	64.21	36.13	6.35	3
142	PD	Male	False	16	56.00	11.37	0.76	0.00	0.00	0
143	PD	Male	True	16	72.00	114.05	17.52	9.66	1.37	1
144	PD	Female	False	16	63.00	0.00	0.00	0.00	0.00	0
145	PD	Male	False	18	68.00	229.91	34.62	55.16	7.03	2
146	PD	Female	False	16	65.00	4.95	0.28	19.55	2.14	0
147	PD	Female	False	18	68.00	45.25	5.94	3.94	0.41	4
148	PD	Male	False	18	65.00	11.96	1.18	0.00	0.00	0
149	PD	Male	False	14	76.00	10.05	1.21	0.00	0.00	0
150	PD	Male	False	14	58.00	0.00	0.00	3.79	0.25	0
151	PD	Male	False	16	54.00	24.73	3.97	0.00	0.00	0
152	PD	Female	False	16	72.00	0.00	0.00	0.00	0.00	4
153	PD	Male	False	20	57.00	10.63	1.11	0.00	0.00	0
154	PD	Male	False	20	71.00	17.15	3.21	0.00	0.00	0
155	PD	Male	False	12	59.00	11.32	1.76	0.00	0.00	0
156	PD	Male	False	20	54.00	76.96	14.31	1.12	0.16	0
157	PD	Female	False	14	71.00	15.56	1.66	32.06	3.42	0
158	PD	Male	False	20	64.00	407.13	62.90	23.01	3.30	0
159	PD	Male	False	18	65.00	4.14	0.22	0.00	0.00	0
160	PD	Female	False	18	65.00	16.94	2.62	0.00	0.00	0
161	PD	Female	False	18	59.00	33.96	5.75	33.04	2.29	0
162	PD	Male	False	18	74.00	21.90	2.51	0.00	0.00	0
163	PD	Female	False	19	67.00	19.12	1.82	19.73	2.60	0

164	PD	Female	False	16	70.00	24.10	1.16	0.00	0.00	0
165	PD	Female	False	16	62.00	73.93	8.92	7.12	0.70	0
166	PD	Female	False	16	58.00	3.32	0.30	0.00	0.00	1
167	PD	Male	False	18	66.00	80.83	7.37	37.98	2.23	0
168	PD	Male	True	16	70.00	23.47	1.86	2.02	0.16	0
169	PD	Female	False	18	52.00	132.21	22.41	0.00	0.00	0
170	PD	Male	False	18	68.00	29.28	6.21	5.66	0.63	3
171	PD	Male	True	12	63.00	0.00	0.00	0.00	0.00	0
172	PD	Male	False	15	75.00	12.28	1.22	0.00	0.00	0
173	PD	Female	False	11	54.00	0.00	0.00	0.00	0.00	0
174	PD	Male	False	13	60.00	14.62	1.33	22.73	2.62	0
175	PD	Male	False	19	62.00	67.99	4.77	0.00	0.00	0
176	PD	Male	False	18	67.00	0.00	0.00	0.00	0.00	4
177	PD	Male	False	15	62.00	2.81	0.28	0.00	0.00	4
178	PD	Male	False	16	72.00	44.98	4.20	38.38	3.88	3
179	PD	Female	False	14	69.00	8.09	1.20	0.00	0.00	0
180	PD	Male	False	18	63.00	555.58	96.28	33.51	5.21	1
181	PD	Female	False	16	54.00	99.96	22.01	0.00	0.00	0
182	PD	Male	False	18	79.00	13.82	0.93	20.54	4.27	1
183	PD	Female	False	18	77.00	0.00	0.00	0.00	0.00	0
184	PD	Male	False	16	55.00	8.52	0.85	30.14	5.21	0
185	PD	Female	False	18	69.00	7.62	0.88	0.00	0.00	0
186	PD	Female	False	16	77.00	0.00	0.00	0.00	0.00	0
187	PD	Female	False	16	59.00	4.07	0.34	0.41	0.05	0
188	PD	Female	True	12	80.00	35.02	2.98	8.29	0.68	0
189	PD	Female	False	18	69.00	0.00	0.00	0.00	0.00	4
190	PD	Male	False	16	69.00	0.00	0.00	0.00	0.00	0
191	PD	Male	False	12	69.00	26.32	1.54	0.00	0.00	4
192	PD	Female	False	14	80.00	182.85	20.13	45.89	6.23	1
193	PD	Male	False	13	59.00	35.76	4.22	0.00	0.00	0
194	PD	Male	False	16	75.00	0.00	0.00	0.00	0.00	4
195	PD	Female	False	14	57.00	19.59	2.02	0.00	0.00	0
196	PD	Male	False	18	74.00	4490.78	1154.13	60.32	4.69	1
197	PD	Male	False	18	70.00	201.21	27.67	0.00	0.00	0

198	PD	Female	True	16	71.00	4384.64	901.04	142.69	16.95	0
199	PD	Female	False	20	77.00	13.83	1.27	0.00	0.00	0
200	PD	Male	False	16	65.00	8.23	0.45	0.00	0.00	0
201	PD	Male	False	18	74.00	187.24	15.78	3.85	0.32	4
202	PD	Female	False	16	54.00	7.15	0.35	0.00	0.00	1
203	PD	Female	False	18	50.00	0.00	0.00	0.00	0.00	0
204	PD	Female	False	20	55.00	40.55	9.22	0.00	0.00	0
205	PD	Male	False	18	74.00	3.64	0.13	0.00	0.00	1
206	PD	Male	False	14	65.00	9.55	0.74	0.00	0.00	0
207	PD	Male	False	18	76.00	0.00	0.00	0.00	0.00	2
208	PSP	Female	False	12	72.00	1540.61	581.89	480.18	76.40	0
209	PSP	Male	True	10	71.00	0.00	0.00	0.00	0.00	0
210	PSP	Male	True	18	58.00	0.00	0.00	0.00	0.00	0
211	PSP	Male	True	12	56.00	0.00	0.00	0.00	0.00	4
212	PSP	Male	True	12	72.00	0.00	0.00	50.59	16.29	0
213	PSP	Male	False	12	56.00	2339.34	1093.41	262.56	72.65	0
214	PSP	Male	True	20	71.00	10.56	2.99	53.03	16.28	3
215	PSP	Male	False	20	70.00	0.00	0.00	0.00	0.00	0
216	PSP	Female	False	16	72.00	0.00	0.00	0.00	0.00	1
217	PSP	Female	False	16	66.00	11.74	1.35	55.46	26.30	0
218	PSP	Female	True	16	64.00	0.00	0.00	0.00	0.00	0
219	PSP	Female	True	19	59.00	288.40	164.91	0.00	0.00	0
220	PSP	Female	False	16	68.00	59.56	26.04	0.00	0.00	1
221	PSP	Male	False	12	68.00	227.69	90.53	87.52	21.84	2
222	PSP	Female	False	12	64.00	9.07	1.28	32.81	2.97	0
223	PSP	Female	False	15	72.00	11.25	2.11	75.38	15.20	0
224	PSP	Female	False	15	71.00	44.69	8.89	65.87	12.89	0
225	PSP	Female	False	16	68.00	0.00	0.00	58.53	1.30	0
226	PSP	Female	False	14	60.00	0.00	0.00	39.49	5.90	0
227	PSP	Female	True	16	63.00	0.00	0.00	155.77	23.57	4
228	PSP	Male	True	18	75.00	0.00	0.00	0.00	0.00	0
229	PSP	Female	False	18	65.00	0.00	0.00	64.98	10.51	1
230	PSP	Female	False	19	72.00	18.51	6.31	112.82	17.52	3
231	PSP	Male	True	14	72.00	70.30	11.02	207.12	23.09	4

232	PSP	Male	False	16	67.00	603.26	166.08	104.81	24.00	0
233	PSP	Female	False	12	78.00	0.00	0.00	32.11	3.07	0
234	PSP	Male	False	16	58.00	17.12	2.93	117.12	23.41	1
235	PSP	Male	False	12	77.00	10.73	0.29	52.67	4.95	3
236	PSP	Male	False	16	65.00	720.76	132.26	156.15	15.01	0
237	PSP	Male	True	16	74.00	21.00	6.02	98.61	10.14	0

Supplementary Table 5 p-values of tests on normal distribution for aSyn and Tau aggregates in CSF

		PD	DLB	AD	PSP	N
aSyn	Shapiro Wilk	0	$7.37*10^{-8}$	$2.44*10^{-7}$	$1.81*10^{-9}$	$9.16*10^{-14}$
	Lilliefors	$7.91*10^{-57}$	$5.73*10^{-9}$	$13.85*10^{-6}$	$1.20*10^{-13}$	$5.24*10^{-26}$
	Kolmogorov Smirnov	$5.72*10^{-18}$	0.0027	0.018	$7.76*10^{-5}$	$8.45*10^{-9}$
	Anderson Darling	$4.18*10^{-76}$	$2.35*10^{-12}$	$5.94*10^{-9}$	$3.00*10^{-17}$	$1.59*10^{-33}$
Tau	Shapiro Wilk	0	$9.03*10^{-7}$	$5.54*10^{-5}$	$4.72*10^{-6}$	$6.85*10^{-12}$
	Lilliefors	$6.56*10^{-57}$	$1.40*10^{-8}$	$3.70*10^{-5}$	$9.74*10^{-4}$	$9.8*10^{-21}$
	Kolmogorov Smirnov	$5.4*10^{-18}$	0.004	0.036	0.103	$4.03*10^{-7}$
	Anderson Darling	$2.12*10^{-69}$	$3.60*10^{-9}$	$4.97*10^{-6}$	$1.35*10^{-5}$	$4.42*10^{-25}$

Supplementary Table 6 Pearson coefficient of correlation values for analysis between aSyn and Tau aggregate concentrations and age, education, sex, disease duration, and death

		All cohorts	PD	DLB	AD	PSP	N
aSyn	Age	0.096	0.157	0.026	0.049	-0.256	0.130
	Education	-0.074	-0.133	0.025	0.183	-0.268	0.009
	Sex	-0.081	-0.061	-0.210	0.179	-0.134	-0.155
	Disease duration	0.093	0.012	0.073	0.130	-0.115	–
	Deceased	0.095	0.226*	-0.225	-0.235	-0.248	0.087
Tau	Age	0.070	0.166	0.031	-0.024	0.016	0.074
	Education	-0.144*	-0.182	-0.001	0.150	-0.258	-0.060
	Sex	-0.043	-0.081	-0.195	-0.060	-0.006	-0.089
	Disease duration	0.068	-0.006	0.106	0.047	0.055	–
	Deceased	0.070	-0.193*	-0.181	-0.357	-0.211	0.085

* indicates a significant correlation with p-values between 0.01–0.05

Supplementary Figure 1 TEM image (a) and size distribution (b) of silica nanoparticles

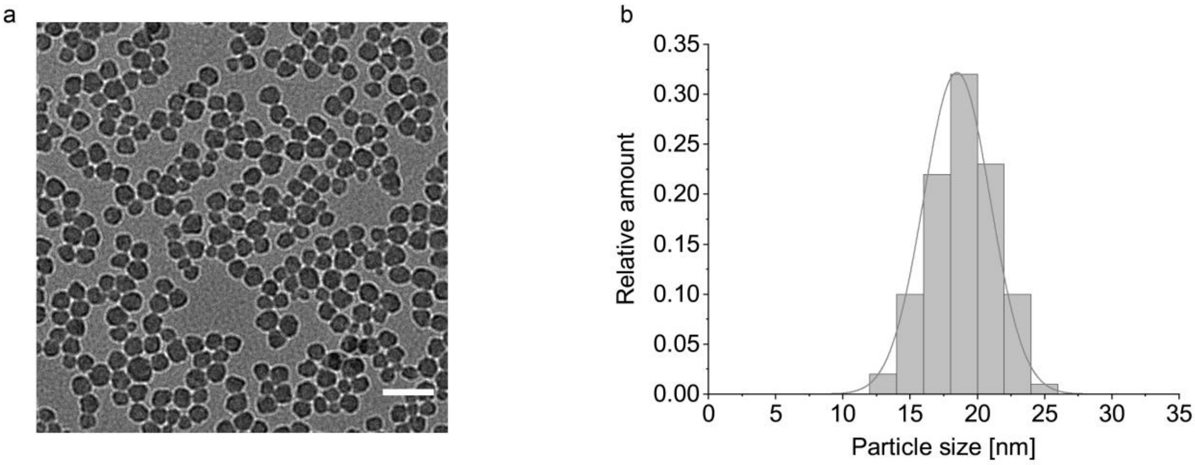

The exemplary image of an aSyn silica nanoparticle (a) shows the typical particle shape of the calibration standard. The scale bar is 50 nm. Aminated silica nanoparticles, which are the basis for peptide conjugation, show a normally distributed particle size with a mean of 18.5 nm (b, normal distribution analysed using Kolmogorov Smirnoff).

Supplementary Figure 2 aSyn (a) and Tau (b) aggregate concentrations grouped by blood-contamination levels

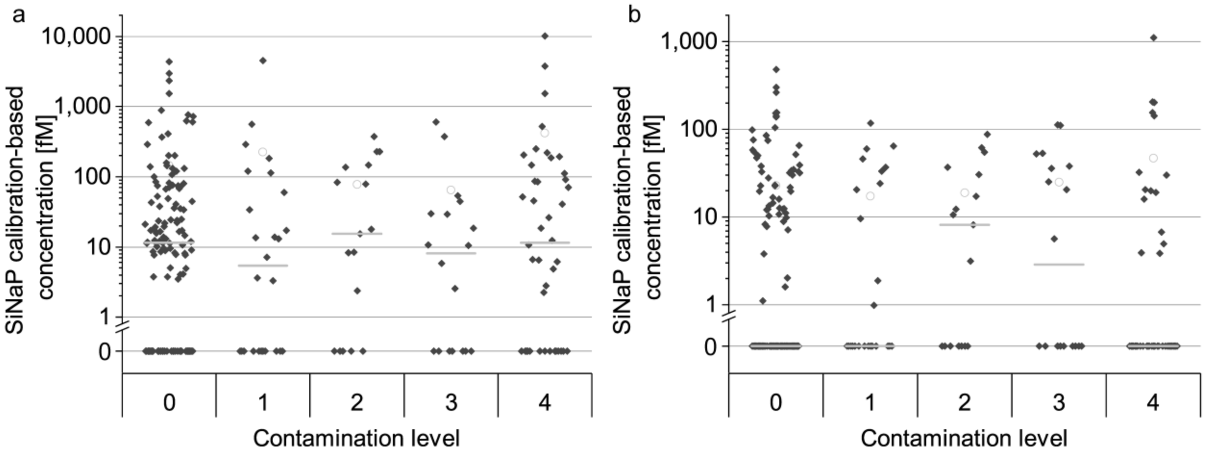

Blood contamination in CSF samples were measured with Combur10 stripes and grouped in 5 contamination levels (0: no contamination, 1: ~10 Ery/µL, 2: ~25 Ery/µL, 3: ~50 Ery/µL, 4: >250 Ery/µL). Higher contamination levels did not result in significant differences in aggregate concentrations (Kruskal-Wallis ANOVA: $p = 0.78$ for aSyn aggregates and $p = 0.63$ for Tau aggregates).
